# DEPRESSION, ANXIETY, AND BINGE EATING BEFORE AND AFTER BARIATRIC SURGERY: PROBLEMS THAT REMAIN

**DOI:** 10.1590/0102-672020180001e1356

**Published:** 2018-06-21

**Authors:** Graziela Aparecida Nogueira de Almeida RIBEIRO, Helenice Brizolla GIAPIETRO, Lídia Barbieri BELARMINO, Wilson SALGADO-JUNIOR

**Affiliations:** 1 Service of Bariatric Surgery, Hospital das Clínicas, Medical School of Ribeirão Preto, University of São Paulo, Ribeirão Preto, São Paulo, SP, Brazil

**Keywords:** Bariatric surgery, Depression, Anxiety, Binge-eatingdisorder, Evaluation., Cirurgia bariátrica, Depressão, Ansiedade, Transtorno da compulsão alimentar, Avaliação.

## Abstract

**Background:**

As the number of surgeries increases and the elapsed time of the realization increases as well, the postoperative evaluations would become increasingly necessary.

**Aim:**

To assess the psychological profile before and after surgery.

**Methods:**

Were evaluated 281 patients from the public service of bariatric surgery. In this study, 109 patients completed the evaluations before surgery (T0) and up to 23 months after surgery (T1); 128 completed the evaluations in T0 and between 24 months and 59 months after surgery (T2); and 44 completed the evaluations in T0 and 60 months after surgery (T3). A semi-structured interview, the Beck Depression Inventory (BDI), Beck Anxiety (BAI), and the Binge Eating Scale (BES) were used.

**Results:**

There was a higher prevalence of female (83%), patients with less than 12 years of education (83%), and patients who have a partner (64%). Analyzing all times of evaluation, regarding anxiety, depression, and binge eating, there was a reduction in all symptoms in T1, pointing to significant improvements in the first 23 months after surgery. Already, in T2 and T3, there was an increase in all indicators of anxiety, depression, and binge eating pointing to the transient impact of weight loss or bariatric surgery on these symptoms.

**Conclusions:**

This study shows the importance of the continuous psychological evaluation and needs for the appropriate interventions for these patients who have undergone bariatric surgery, even after weight loss.

## RESUMO

**Racional:**

Avaliações pós-operatórias têm se tornado necessárias em ciruriga bariátrica considerando o grande número de operações e o tempo decorrido de sua realização.

**Objetivo:**

Avaliar o perfil psicológico de pacientes de um serviço público de cirurgia bariátrica antes e após o procedimento.

**Métodos:**

Foram avaliados no total 281 pacientes. Destes, 109 completaram as avaliações antes (T0) e até 23 meses após a operação (T1); 128 completaram as avaliações em T0 e entre 24 meses e 59 meses após a operação (T2); e 44 completaram as avaliações em T0 e 60 meses após a operação (T3). Foram utilizados entrevista semi-estruturada, Inventário Beck de Depressão (BDI), Inventário Beck de Ansiedade (BAI) e Escala de Compulsção Alimentar Periódica (ECAP).

**Resultados:**

Observou-se maior prevalência de mulheres (83%), pacientes com menos de 12 anos de escolaridade (83%) e pacientes que tinham um companheiro(a) (64%). Ao analisar todos os tempos de avaliação, observou-se que, com relação à ansiedade, depressão e compulsão alimentar, houve redução de todos os sintomas em T1, apontando para melhorias significativas nos primeiros 23 meses após a operação. Já em T2 e T3 observou-se aumento de todos os indicadores de ansiedade, depressão e compulsão alimentar, apontando para o impacto transitório da perda de peso alcançada pela cirurgia bariátrica nesses sintomas.

**Conclusões:**

Este estudo mostra a importância de avaliações psicológicas contínuas e a necessidade de intervenções multiprofissionais apropriadas para pacientes submetidos à cirurgia bariátrica, mesmo após a perda de peso.

## INTRODUCTION

Bariatric surgery has been considered the treatment of choice for obesity grade 3 or for obesity grade 2 with comorbidities^4^ ^,^ ^12^ ^,^ ^22^. As the number of surgeries increases and the elapsed time of the realization increases as well, the clinical and psychological postoperative evaluations would become increasingly necessary, considering the importance of verifying also under the psychosocial perspective, if symptoms are changing as a result of weight loss^1^ ^,^ ^13^.

Studies have suggested a substantial percent of patients presenting for bariatric surgery report depression and anxiety symptoms, as well as eating behavior changes^16^ ^,^ ^21^. However, the evaluations conducted after surgeries have shown divergent results, as they have been conducted at different times postoperatively. Studies assessing symptoms of depression, anxiety, and eating disorders one or two years after surgery show a significant improvement of symptoms^6^ ^,^ ^14^ ^,^ ^20^. Other studies conducted after a longer time (i.e., 4-5 years) show that improvements begin to decrease and, in some cases, deterioration of these diseases may occur^5^ ^,^ ^7^ ^,^ ^19^.

It is also observed that most of the studies that propose to evaluate the psychosocial aspects before and after bariatric surgery make evaluations prior to surgery and then, only look at data at one specific time point after surgery, usually a few years postoperatively^6^ ^,^ ^14^ ^,^ ^20^. Therefore, the importance and necessity of studies that propose to evaluate the psychosocial aspects at different times after surgery have become clear, including a longer period of time after surgery^19^.

This study aimed to evaluate the presence of anxiety, depression, and binge eating indicators both before and after bariatric surgery

## METHODS

In this study, 281 patients were evaluated at Bariatric Surgery Clinic of the Clinical Hospital of the Faculty of Medicine of Ribeirão Preto of the University of São Paulo, SP, Brazil. Of these patients, 109 completed the evaluations before surgery (T0) and up to 23 months after surgery (T1); 128 completed the evaluations in T0 and between 24 months and 59 months (T2); and 44 completed the evaluations in T0 and 60 months (T3). It is important to note that all of the patients underwent Roux-en-Y gastric bypass, which is considered a mixed surgery and is proven to be effective in weight loss and maintenance^13^.

The following instruments were used for this study: a semi-structured interview, which aimed to investigate sociodemographic data and feelings about weight and body size; the Beck Depression Inventory (BDI)^8^and Beck Anxiety Inventory (BAI)^8^, which aimed to investigate the presence of symptoms of depression and anxiety, respectively; and the Binge Eating Scale (BES)^10^ to evaluate the presence of symptoms of binge eating.

Data from preoperative psychological evaluations were collected in the bariatric clinic before undergoing the bariatric surgery. Data from the postoperative evaluations were collected when the patients were returning to the bariatric surgery clinic. Patients were asked to answer the questionnaires while waiting for the outpatient consultation by the physician or dietitian.

In all evaluations, the instruments were administered individually by staff psychologists.

### Statistical analysis

For statistical analysis, the mean values and standard deviation (SD) for age and BMI were calculated using a *t-*test for paired samples. Regarding gender, education, and marital status, frequency and percentage values were calculated in the T0 period. For questions concerning feelings about weight and body size, ANOVA was applied. For the other instruments, the data were codified according to the specific recommendation instrument, proceeding to statistical treatment using ANOVA, too. All of the statistical analyses were performed using the SPSS 17.0 package. *p*<0.05 was considered statistically significant.

## RESULTS

In Table 1, which describes the characteristics of the patients according to gender, education, and marital status, there was a higher prevalence of female patients (83%), patients with less than 12 years of education (83%), and patients who have a partner (64%).

**TABLE 1:** Characteristics of patients by gender, education and marital status

Characteristics	T0 (n=281)	
	F	%
Gender		
Female	233	83
Male	48	17
Education		
Up to 9 years	118	42
From 10 to 12 years	116	41
More than 13 years	47	17
Marital status		
With partner	178	64
No partner	103	36

T0=before surgery

The characterization of patients in relation to age and BMI before and after bariatric surgery is shown in Table 2. Analyzing all times of evaluation, it was observed that the preoperative BMI was significantly higher compared to the postoperative BMI in all times (T1, T2, and T3).

**TABLE 2:** Age and BMI of the participants at all times (mean and SD)

Characteristics	T0 (n=281)		T1 (n=109)		T2 (n=128)		T3 (n=44)	
	M	SD	M	SD	M	SD	M	SD
Age	40.7	9.8	39.6	9.3	41.3	10.5	41.8	9.6
BMI - before surgery	50.9	7.4	49.9*	6.8	51.5*	7.7	52.9*	7.8
BMI - after surgery	--	--	31.9*	8.1	29.4*	9.4	31.8*	9.1

BMI=body mass index; T0=patients who completed the evaluations before surgery; T1=patients who completed the evaluations up to 23 months after surgery; T2=patients who completed the evaluations between 24 months and 59 months after surgery; T3=patients who completed the evaluations 60 months after surgery; n=number of patients; *T test for paired samples. p<0.0001

Table 3 which shows the frequency and percentage of satisfaction or dissatisfaction with weight and body size of the patients throughout the postoperative period indicates a higher percentage of feelings of satisfaction at all times compared to feelings of dissatisfaction. However, when comparing the three different times, it was noted that the comparison between T1 vs. T2 and T1 vs. T3, the percentage of dissatisfaction with weight was significantly different, pointing to a greater sense of satisfaction at T1 compared to T2 and T3 (ANOVA. *p*<0.05 and *p*<0.005. respectively).

**TABLE 3:** Frequency and percentage of satisfaction or dissatisfaction with weight and body size at all times after surgery

Characteristics	T1 (n=109)		T2 (n=128)		T3 (n=44)	
	F	%	F	%	F	%
Feelings about weight and body size						
Good / satisfied	106	97.2*^.)^**	107	83.4*	32	72.1**
Bad / insatisfied	3	2.8	21	16.7	12	27.9

T1=patients who completed the evaluations up to 23 months after surgery; T2=patients who completed the evaluations between 24 months and 59 months after surgery; T3=patients who completed the evaluations 60 months after surgery; n=number of patients. Anova. *p< 0.05; Anova. ** p< 0.005

**FIGURE 1:**
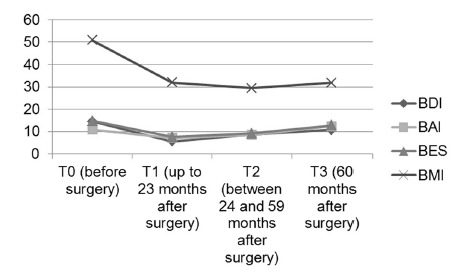
Average BMI and average score on the BDI, BAI and BES in all evaluation times

Figure 1 shows the results for the average scores of patients in BDI, BAI, and BES, as well as the average BMI in all times of evaluation.

Comparing the average scores of BDI, BAI and BES, statistically significant differences in all comparisons between the different times of evaluation were observed. Regarding the BDI, in comparing the assessments made before surgery (T0) with T1, T2, and T3, there were statistically significant differences in all of the comparisons (ANOVA, *p*<0.0001, *p*<0.0001, and *p*<0.03, respectively).

In comparison between T1 vs. T2 and between T2 vs. T3, statistically significant differences (ANOVA, *p*<0.002, *p*<0.003, respectively) were also observed.

Regarding the BAI, the comparison of the evaluations T0 with T1, T2, and T3 showed significant differences in comparison with T1 (ANOVA, *p*<0.0001) and T2 (ANOVA, *p*<0.05). In addition, significant differences were observed when comparing T1 vs. T3 (ANOVA, *p*<0.003) and T2 vs. T3 (ANOVA, *p*<0.05).

Regarding the BES, when the T0 evaluations were compared with the different times, statistically significant differences were observed between T0 vs. T1 (ANOVA, *p*<0.0001), T0 vs. T2 (ANOVA, *p*<0.0001), and T1 vs. T3 (ANOVA, *p*<0.01).

In the T0, 45% of patients had indicative scoring of anxiety symptoms (mild, moderate, or severe), 58% had indicators of depressive symptoms (mild, moderate or severe), and 32% had indicators suggesting binge eating (moderate or severe). In the T1, only 20% of patients had scores suggestive of some degree of anxiety, 13% had a score indicative of a degree of depression, and 11% had a score indicative of binge eating.

In the T2, it was observed a still small percentage of patients with scores indicating pathological presence of symptoms of anxiety, depression and binge eating: 33% of patients with indicators suggestive of anxiety, 27% with depression indicators, and 16% with binge-eating indicators. However, it is noteworthy that the score in the different instruments begins to increase.

At time T3, there was a significant increase in scoring in the different instruments as follows: 40% of patients had the presence of some degree of anxiety indicators, 35% of patients had indicators of depression symptoms, and 27% had binge-eating indicators.

## DISCUSSION

This study aimed to evaluate psychosocial symptoms in patients of bariatric surgery at different times before and after surgery. As far as can be seen, this is the first study that evaluates during different times pre- and postoperative period, including a wide range of times, the psychosocial symptoms of patients who underwent Roux-en-Y gastric bypass surgery.

Considering the characteristics of the sample, it was observed that most of them were female, which is consistent with other studies that point to a higher prevalence of surgical treatment for obesity among women. It can be suggested here in Brazil, as well as in other countries like the United States and Canada, that women seek more health services compared to men, mainly because they have a greater concern for their health^3^. One can also think that this higher prevalence is justified due to the search for an ideal of beauty and positive attributes, both associated with thinness, accruing from the many social demands that fall on women^1.15.22)^.

Comparing the BMI before and after surgery, the results point to a significant weight loss at all times (T1, T2 and T3). However, when comparing different times after surgery, it is observed that the greatest amount of weight loss is concentrated until 23 months. After that, the tendency is that the weight is stabilized between 23 and 59 months. After 60 months, the weight increases gradually. Note that it not only weight loss but also the maintenance of weight loss appears to be significant challenges to be achieved by the operated patients. In terms of BMI, although the patients have not returned to their pre-operative BMI, which shows the favorable results of the surgery, follow up appointments and care throughout life to treat this chronic disease^9.11.17)^are necessary.

Regarding anxiety, depression, and binge eating, there was a reduction in all symptoms in T1, pointing to significant improvements in the 1^st^ 23 months after surgery, as demonstrated in other follow-up studies^2^ ^,^ ^18^. Already, in T2 and T3, there was an increase in all indicators pointing to a worsening of symptoms of anxiety, depression, and binge eating. These results emphasize the importance of seeking to understand more clearly the impact that weight fluctuations have on people that experienced the bariatric surgery. Furthermore, the results point to the importance of appropriate interventions over time, even after weight loss.

The results regarding satisfaction with weight and body size draw attention. The higher prevalence of feelings of satisfaction is observed in T1 (i.e., the first two years after surgery) even when patients were experiencing a fast weight loss and changes in their size and body shape. However, over time, when the weight loss started to decrease (T2), it is possible to observe a decrease in feelings of satisfaction. In T3, this difference is even greater. The reduction of weight loss (as occurred in T2) and/or weight gain (as occurred in T3), as well other changes (internal and external) experienced by these people have probably contributed to this variation. In addition to feelings of dissatisfaction, it is clear that the indicators of the levels of depression, anxiety, and binge eating go in parallel. The presence of these indicators confirms the hypothesis that dissatisfaction resulted not just because of body size, but dissatisfaction seems to be generalized and may result in the presence of mental disorders. When considering the physical body, these people have lost a significant amount of weight. These people also have sagging skin in different parts of the body, which in turn contributes to the onset of functional difficulties with walking and hygiene. They may not have been prepared for these other changes to their physical appearance. In addition to the “cosmetic” changes, there are internal changes. Among them, it can mention the perception that the surgery itself and subsequent weight loss did not lead the patient to achieve the changes before desired and often faintly planned. That is, at the end of five years or more, the patients realize that they failed to achieve what they wanted for different reasons, among them the own internal difficulties. When patients are asked about their feelings regarding their current size and body shape, it is clear that the answers are not restricted to the size and shape of their bodies, but the answers also include the way they have been able to deal with their current bodies. If they managed to achieve the different goals pursued previously, the feelings are satisfaction. Otherwise, dissatisfaction becomes even greater.

One can hypothesize that weight changes after surgery influenced the BDI, BAI, and BES values. In the study of Strain^18^ the authors observed that depressive symptoms that are commonly associated with morbid obesity improve after bariatric surgery. Similarly, this study also noted these changes within that period. However, one cannot say that these changes remain stable, as 24 months after surgery, patients returned to increased levels of these symptoms^18^. The literature is replete with studies demonstrating the improvement in psychosocial functioning as a function of weight changes after surgery. However, these changes have been observed in the short-term (i.e., up to two years of surgery)^6.22)^. On the other hand, a study evaluating psychosocial functioning after a longer time after surgery showed that significant improvements remained at the 4-year period^6^.

One of the limitations of this study includes the fact that the postoperative evaluations were made from available samples. In other words, the patients were evaluated when they returned to medical, nutritional, and psychological counseling in the clinic. A strong point of the study refers to the fact of having a prospective, long-term, covering rating on a comprehensive *continuum* of time.

## CONCLUSION

This study shows clearly the importance of the continuous evaluation and need for the appropriate interventions for these patients who have undergone bariatric surgery, even after weight loss. It is important to consider the need for continued treatment, emphasizing the idea that because obesity is a chronic disease, it requires multidisciplinary and long-term treatment, even for surgical cases.
